# Why You Should Report Bayes Factors in Your Transcranial Brain Stimulation Studies

**DOI:** 10.3389/fpsyg.2018.01125

**Published:** 2018-07-02

**Authors:** Anna Lena Biel, Elisabeth V. C. Friedrich

**Affiliations:** Biological Psychology, Department of Psychology, Ludwig-Maximilians-Universität München, Munich, Germany

**Keywords:** Bayesian statistics, null results, reproducibility, tACS, TBS, tDCS

In this commentary, we argue that it is essential to determine whether a non-significant sample effect really indicates that a particular application of transcranial brain stimulation (TBS) had no effect. We point out that non-significant results do not necessarily support a non-effect and show why reporting Bayesian statistics can help answering whether there is good enough evidence for the null hypothesis in your TBS data.

TBS aims to modulate or probe neural activity. However, reports on physiological and behavioral changes often failed to show conclusive results (Hill et al., 2016; Mancuso et al., 2016). There are many possible reasons for such inconsistencies. Recently it has been demonstrated that sufficiently large samples are essential in designing TBS experiments (Minarik et al., 2016). However, a-priori power-analyses are often skewed due to publication bias, where large or statistically significant effects get published more often. Therefore, the actual efficacy of TBS might be overestimated. While it is possible to adjust overestimated effect size for publication bias, insights about ineffective TBS duration, intensity, frequency or montage cannot be taken into account when unpublished. Thus, initiatives such as this Research Topic should encourage researchers to publish their non-significant outcomes in order to make relevant contributions to the field as well.

However, conventional significance testing cannot determine whether non-significant outcomes really indicate that a TBS protocol had no effect. In conventional significance testing, a research hypothesis assuming a certain population effect (H1), is compared against the null hypothesis assuming a non-effect in the population (H0). The probability for getting an observed sample effect is evaluated based on the significance level. If the outcome is below-threshold, one can provide evidence *against* the null hypothesis and accept the research hypothesis – whereas it is never possible to state evidence *for* the null hypothesis.

Bayes factors (BFs) are a powerful tool for evaluating evidence both for the research hypothesis and for the null hypothesis (e.g., Rouder et al., 2009; Dienes, 2011; Kruschke, 2011). In case of a conventional non-significant test, the observed sample effect either truly supports the null hypothesis or was too weak to yield evidence against it. Bayes factor tests, however, are highly useful to inform whether the data do or do not favor the null hypothesis over the alternative. We demonstrate this by simulating a series of fictional TBS experiments.

We assumed that *N* participants performed a task under two conditions, namely sham and real TBS. Task performance in these TBS conditions would differ by a true population effect *dz*. This difference in task performance was simulated by selecting *N* observations from a normal distribution with a mean of *dz* and a standard deviation of 1. We repeated this fictional experiment 1000 times. Each time, we tested for the effect of condition by comparing the research hypothesis assuming an increase of task performance during real TBS relative to sham TBS conditions (H1: *dz* > 0), against the null hypothesis assuming a non-effect (H0: *dz* = 0). First, we calculated a one-sided one-sample *t*-test, which is conventionally considered as significant (i.e., H0 is rejected) if *p*-values fall below 0.05. Next, we calculated a corresponding Bayes factor test which yields a *BF* quantifying how well H1 predicts the empirical data relative to H0 (*BF*_10_). Here, BFs above 1 indicate evidence for H1 over H0, whereas BFs below 1 suggest the exact opposite. By convention (Jeffreys, 1961; Lee and Wagenmakers, 2014), the strength of evidence for one hypothesis compared to its competing hypothesis is regarded as noteworthy if BFs are above 3 or below 0.33. Thus, BFs between 0.33 and 3 are considered as inconclusive, or only anecdotal evidence for any hypothesis. We conducted this simulation for eight samples differing in sample size (*N* = 10, 20, 30, 40, 50, 60, 70, or 80) and six TBS protocols differing in population effect size compared to sham (*dz* = 0, 0.1, 0.2, 0.3, 0.4, or 0.5). The simulation was run using R (version 3.2.4; R Core Team, 2016) where BFs were computed using default priors by the R package BayesFactor (version 0.9.12-2; Morey and Rouder, 2015), modeling H1 as a Cauchy distribution scaled in standardized effect sizes with scale factor = 0.7 Cohen's *dz* units.

Figure 1 depicts *p*-values and Bayes factors obtained from two exemplary population effect sizes (Figure 1A: *dz* = 0.2, Figure 1B: *dz* = 0.5). Unsurprisingly, with increasing sample size, more *t*-tests were significant (*p* > 0.05) and more corresponding Bayes factors indicated at least moderate evidence for H0 over H1 (*BF* < 0.33). Similarly, fewer *t*-tests were non-significant and fewer Bayes factors favored the H0 with increasing population effect size.

**Figure 1:**
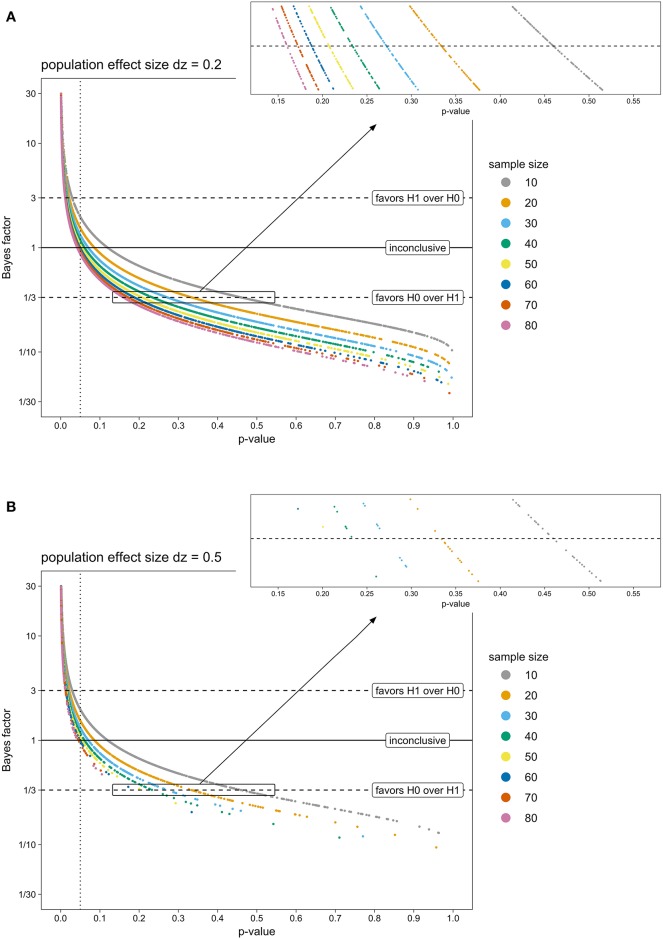
*P*-values from a one-sided one-sample *t*-test and corresponding Bayes factors of simulated TBS experiments, for eight sample sizes (colored points) and two exemplary population effect sizes (**A**: *dz* = 0.2; **B**: *dz* = 0.5). *T*-tests with a *p*-value below 0.05 (dotted line) are conventionally considered as significant and H0 is rejected. BFs above 3 (upper dashed line) indicate evidence for H1 being more likely than H0. BFs below 0.33 (lower dashed line) yield evidence for H0 being favored over H1. BFs between 0.33 and 3 (area between the two dashed lines) are considered as inconclusive, or not more than anecdotal evidence for one of the hypotheses. *Note. BF, Bayes factor; H0, null hypothesis; H1, research hypothesis; TBS, Transcranial Brain Stimulation*.

Interestingly, critical *p*-values, where corresponding BFs fell below 0.33 (i.e., indicating at least moderate evidence for H0 over H1), decreased when sample size increased. For example, for samples of 10 participants, *p*-values as high as 0.45 were associated with BFs being inconclusive (0.33 > *BF* > 1). Only *p*-values beyond 0.45 were indicative for at least moderate evidence supporting H0 (*BF* < 0.33). In contrast, for samples of 80 participants, tests with *p*-values around 0.15 or more could be considered to favor H0 according to BFs. Thus, when small samples were tested, non-significant *p*-values had to be much larger for corresponding BFs to indicate at least moderate evidence for H0 than in the case of larger samples.

This relation between *p*-values, BFs and sample size stayed the same across population effect sizes: Population effect size only influenced how many *t*-tests were non-significant or BF-tests favored the H0 overall, but did not influence the range of non-significant *p*-values where corresponding BFs remained inconclusive.

In line with these described observations from simulated TBS experiments, similar associations between *p*-values and BFs have been established for other statistical tests and other models of H1 (e.g., Dienes, 2014, 2015). Taken together, they illustrate the following: First, non-significant tests with a high *p*-value do not automatically prove the null hypothesis to be true, but might indicate inconclusive evidence. Second, sample size heavily influences the threshold of critical *p*-values where Bayes factors indicate that the null hypothesis is more likely than the research hypothesis.

Thus, we conclude that any non-significant findings from conventional significance testing should be supported with evidence from Bayes Factor analyses. This is especially essential in the case of small samples. Of course, Bayesian alternatives to conventional hypothesis testing are not restricted to this case but may be advantageous in many situations. Without entering the debate whether inferential decisions should be based on a purely Bayesian approach (e.g., Dienes and Mclatchie, 2018), we argue that Bayes factor tests may be highly useful for the TBS community by distinguishing between evidence for an (un-)successful TBS protocol and inconclusive evidence. The approach of using Bayes factors to get the most out of non-significant results (Dienes, 2014) is therefore most attractive for the field: Showing the absence of a particular effect of TBS by means of Bayes factor tests may impact on the choice of stimulation parameters more positively than merely reporting conventional non-significant tests.

## Practical recommendations

- The absence of a particular effect of TBS compared to sham TBS can be demonstrated by reporting Bayes factors favoring H0 (there is no condition difference between sham and real TBS) over H1.
- Similarly, the specificity of an observed TBS effect can be shown by reporting Bayes factors favoring H0 (the control condition does not differ from zero) over H1.
- For non-significant *t*-tests, corresponding Bayes factors for *p*-values as high as 0.45 may indicate inconclusive evidence for either H0 or H1 when testing small samples around 10 subjects.
- For other standard statistical tests (*t*-tests, ANOVAs, regressions, etc.), there is easy-to use open-source software (JASP Team, 2018) available, providing both conventional tests as well as their Bayesian alternatives.

## Data availability statement

The raw data supporting the conclusions of this manuscript will be made available by the authors, without undue reservation, to any qualified researcher.

## Author contributions

Both authors were involved in the conceptualization of the topic. AB conducted the simulation, and prepared and wrote the manuscript. EF edited the manuscript.

### Conflict of interest statement

The authors declare that the research was conducted in the absence of any commercial or financial relationships that could be construed as a potential conflict of interest.
